# Preoperative Concentrated Urine Increases the Incidence of Plasma Creatinine Elevation After Major Surgery

**DOI:** 10.3389/fmed.2021.699969

**Published:** 2021-07-19

**Authors:** Dominique Engel, Lukas M. Löffel, Patrick Y. Wuethrich, Robert G. Hahn

**Affiliations:** ^1^Department of Anesthesiology and Pain Medicine, Inselspital, Bern University Hospital, University of Bern, Bern, Switzerland; ^2^Research Unit, Södertälje Hospital, Södertälje, Sweden; ^3^Karolinska Institutet at Danderyds Hospital (KIDS), Stockholm, Sweden

**Keywords:** creatinine plasma urine, acute kidney injury physiology, dehydration urine, general surgery, osmolality urine, fluid retention

## Abstract

**Background:** Postoperative elevation of plasma creatinine is a frequent complication to major surgery. A rise by 50% fulfills the criterion for Acute Kidney Injury. We studied the relationship between concentrated urine before surgery, which is usually a sign of chronically low intake of water, and the perioperative change in plasma creatinine.

**Methods:** The creatinine concentration was measured in plasma and urine just before and at 6 h, 1 day, and 2 days after major abdominal surgery in a consecutive series of 181 patients. Receiver operating curve analysis was used to find the optimal cut-off to separate concentrated from diluted urine.

**Results:** Urine creatinine of 11.3 mmol/L before the surgery started was exceeded in one third of the patients and associated with greater increase in plasma creatinine at 6 h (median 21 vs. 10%) and at 1 day postoperatively (21 vs. 7%; *P* < 0.0001). Elevation of plasma creatinine of >25% occurred in 41% and 19% in those with high and low urine creatinine, respectively (*P* < 0.001) and an increase by >50% in 16% and 10% (*P* = 0.27). Patients with high urine creatinine before surgery failed to further concentrate their urine during the perioperative period, which is normally associated with intensified renal fluid conservation.

**Conclusion:** High urinary concentration of creatinine before surgery should be considered as a risk factor for postoperative elevation of plasma creatinine. The mechanism is probably that the renal threshold is then more easily reached.

## Background

Postoperative acute kidney injury (AKI) is a complication to major surgery that is most commonly diagnosed by measuring plasma creatinine. The consensus criteria for Stage 1 AKI are fulfilled if the increase in plasma creatinine amounts to >26.5 μmol/L within 48 h after surgery, or an increase by 50% compared to baseline, known or presumed to have occurred within the prior 7 days. Alternatively, the urine flow is below 0.5 mL/kg/h for 6 h (KDIGO criteria) (1). Chronic kidney injury occasionally ensues, and follow-up studies show an increased overall morbidity and mortality (2, 3).

The background is probably multifactorial. Factors that are statistically associated with increasing plasma creatinine include arterial hypotension (4–6), restrictive fluid therapy (3, 7) and a high body mass index (8), while opinions differ regarding the role of intraoperative oliguria (9, 10).

Plasma creatinine increases permanently when 50% of the renal function is lost, but the fact that transient elevation of plasma creatinine is common after major surgery raises the question whether other mechanisms than injury to kidney cells may be involved.

In the present study, we propose an alternative explanation to these events that does not involve injury to kidney cells. The question is why plasma creatinine increases at all, since the dilution effects of the perioperative fluid therapy would rather decrease the concentration. Specifically, we examined whether concentrated urine before surgery predisposes to elevation of plasma creatinine after major surgery. Concentrated urine implies that the urinary creatinine is high, whereby less surgery-induced renal water conservation would be required to surpass the renal threshold for concentrating creatinine.

Biomarkers of concentrated urine have been used to study dehydration in sports medicine (11–13) and include creatinine, osmolality, and urine-specific weight. These three biomarkers are frequently elevated in the general population (14), where concentrated urine reflects a chronically low daily intake of water (15, 16). All three were measured by us to study whether creatinine undergoes concentration or dilution changes during surgery that are specific to this biomarker.

Our hypothesis was that concentrated urine before surgery increases the risk of occurrence of a postoperative elevation of plasma creatinine.

## Methods

### Ethical Approval

Ethical approval for this study was provided by the Ethical Committee of the Canton Bern, Switzerland (KEK Bern, Project-ID 2018-01804, Chairperson Professor C. Seiler on December 3rd, 2018) and registered at ClinicalTrials.gov as NCT03788070. Written informed consent was obtained from all patients before initiation of the study. This single-center prospective observational study accorded with the Strengthening the Reporting of Observational Studies in Epidemiology (STROBE) recommendations and the Declaration of Helsinki.

### Study Population

Our purpose was to study the influence of concentrated urine on the risk of having a postoperative elevation of plasma creatinine. Data from a consecutive series of patients at the Department of Urology of the University Hospital Bern who were undergoing major abdominal urologic surgery were used. Other research questions included fluid balance, postoperative nausea and gastrointestinal function, and will be reported elsewhere. Inclusion criteria were age >18 years, elective major urologic laparotomy or robotic assisted laparoscopy. Patients with preoperative intravenous administration of fluid and end-stage kidney disease were excluded.

### Experimental Procedure

No enteral bowel preparation was performed preoperatively. Pre-operative oral hydration was allowed up to 2 h before starting the anesthesia. Surgery was performed under general anesthesia, as described elsewhere (5). Fluid administration consisted of Ringer's lactate infused at a rate of 2 mL/kg/h. If hypotension was observed (mean arterial pressure <60 mmHg), norepinephrine was titrated to a maximum of 8 μg/kg^/^h after an initial bolus of 5–10 μg, or a 250 mL fluid bolus of Ringer's lactate was administered in cases where the patient was judged to be hypovolemic. Blood loss of up to 500 mL was replaced with an equal amount of Ringer's lactate. Packed red blood cells were transfused if the blood hemoglobin (Hb) concentration fell below 80 g/L (<100 g/L in patients with severe coronary artery disease). Additional boluses of Ringer's lactate (250 mL) were administered as a rescue medication if the mean arterial pressure was persistently low or if acidosis developed. Patients were allowed to drink clear fluids while in the intermediate care unit. No diuretics were administered during surgery or during the follow-up period.

### Measurements

Urine and plasma samples were collected the day before surgery, directly after induction of anesthesia, at 6 h postoperatively, and at 6 a.m. of the 1st and 2nd postoperative days. Whole blood was analyzed for the Hb concentration and plasma/serum/urine for creatinine and osmolality.

Measurements of osmolality were performed on a Station 6060 TT instrument (Array Global Business Inc., Japan) and all other biochemical analyses on a Cobas 8000/ISE Modul system (Roche Diagnostics, Basel, Switzerland) in the certified clinical chemistry laboratory at University Hospital Bern. Urine specific gravity was determined with a digital photometric refractometer in steps of 0.005 based on the reflection coefficient of light directed to strips dipped in urine (Uricon-Ne, Atago CO, LTD, Tokyo, Japan).

### Statistical Analysis

The results are presented as the median and 25–75th percentile range. Receiver operating curves (ROC) were used to search for cut-off values in urinary creatinine that distinguished patients who showed a postoperative rise in plasma creatinine of 25 and 50% from the others. Continuous data measured in these groups were compared with the Mann-Whitney's test and percentages by using contingency table analysis. Changes of continuous data were evaluated by the Wilcoxon's matched pair-test. *P* < 0.05 was considered statistically significant.

## Results

In total, 193 patients were recruited between February 2019 and March 2020. One withdrew consent, five patients dropped out, and complete data were missing from six patients, making 181 evaluable data sets. The original plan was to include about 200 patients, but the study had to be terminated due to the Covid-19 crisis.

### Analysis Plan

The presentation is focused on postoperative elevation plasma creatinine as a complication of the surgery and investigates the relationship of this elevation to concentrated urine before the operation as the index of low habitual intake of water or dehydration. Three biomarkers of concentrated urine (osmolality, creatinine and specific weight) were measured, but we used urine creatinine as the key variable because this biomarker is used to diagnose AKI.

### Cut-Off for Urine Creatinine

**Figure 1:**
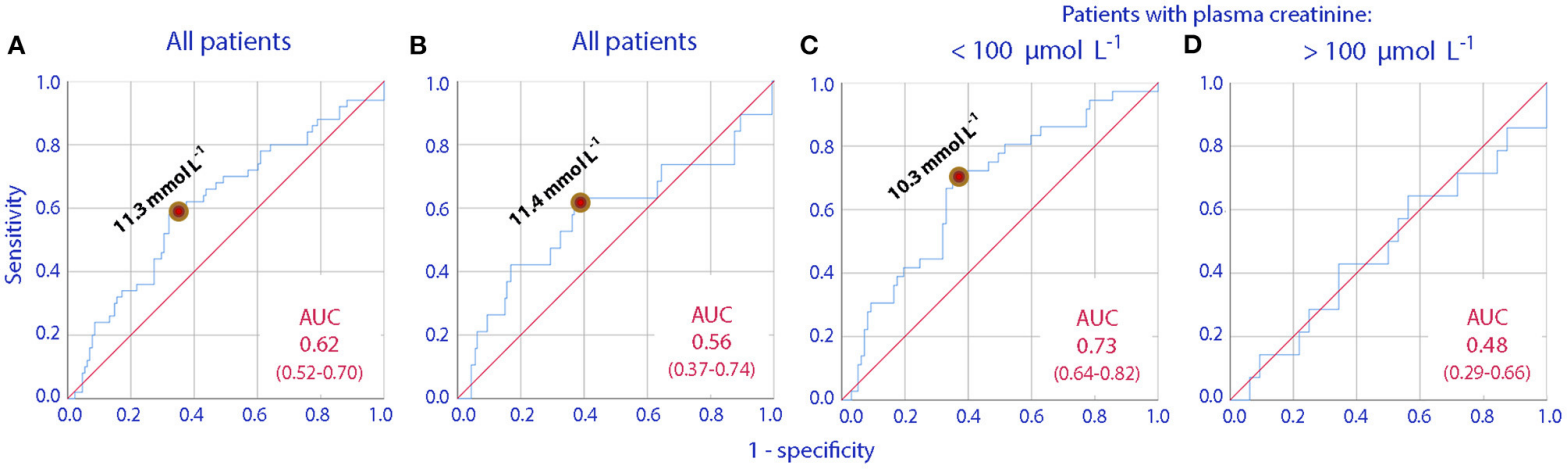
Receiver operating curves (ROC) showing how increasing urinary creatinine concentrations can predict a postoperative increase in plasma creatinine by **(A)** >25% in all patients, **(B)** >50% in all patients, **(C,D)** >25% in patients who have plasma creatinine concentrations lower or higher than 100 μmol/L before the initiation of surgery. The area under the curve (AUC) and its 95% confidence interval are given in each subplot.

ROC curves were used to find if any preoperative urine creatinine concentration could distinguish between patients who would later have an increase in plasma creatinine of >25 and >50% from before surgery to the 1st postoperative day. The optimal cut-off was 11.3 μmol/L, and this value was used for further evaluations (Figures 1A,B).

### Characteristics of Patients With High/Low Urine Creatinine

Table 1 shows demographic and basic laboratory data separately for patients with low (<11.3 μmol/L) vs. high (≥11.3 μmol/L) urine creatinine before surgery.

**Table 1:** Demographic data and biochemistry before surgery.

**Variable**	**U-creatinine <11.3 mmol/L (*N* = 111)**	**U-creatinine ≥11.3 mmol/L (*N* = 70)**	**Significance**
**Demographic data**			
Age (years)	65 (56–69)	64 (58–72)	*p* = 0.44
Body weight (kg)	78 (67–88)	89 (77–100)	*p* < 0.001
BMI (kg/m^2^)	26.2 (23.5–28.9)	28.4 (25.0–32.0)	*p* < 0.002
ASA class I-II/III-V (N)	49 (54%)/61 (68%)	41 (46%)/29 (32%)	*p* = 0.09
Smoking (%)	22	45	*p* < 0.01
Diabetes (%)	10	10	*p* = 0.82
Antidiabetic medication (%)	10	9	*p* = 0.95
Hypertension (%)	42	55	*p* = 0.12
ACE/ARB inhibitor medication (%)	32	37	*P* = 0.62
Diuretic medication (%)	11	6	*p* = 0.35
**Laboratory data**			
Hb start of surgery (g/L)	130 (126–142)	139 (130–145)	*p* < 0.002
S-osmolality (mosmol/kg)	288 (284–290)	288 (283–291)	*p* = 0.70
U-specific weight (no unit)	1.012 (1.009–1.016)	1.015 (1.010–1.020)	*p* < 0.001
U-osmolality (mosmol/kg)	512 (405–646)	650 (540–746)	*p* < 0.001

*Data are the median (25–75th percentiles) and groups compared by using the Mann-Whitney U test, except for incidences, which were compared by contingency table analysis*.

Patients with high urine creatinine had a higher body weight (mean, 89 vs. 78 kg, *P* < 0.001) and were more often smokers (64 vs. 20%; *P* < 0.01).

The laboratory data show that those with high urine creatinine also had higher urine specific weight, 1.015 vs. 1.012, *P* < 0.001) and higher urine osmolality (650 vs. 512 mosmol/kg, *P* < 0.001) than the other patients. By contrast, serum osmolality was the same regardless of urine osmolality.

### Elevation of Plasma Creatinine

Overall, 50 patients (28%) had an increase in plasma creatinine by >25% and 22 patients (12%) an increase in plasma creatinine exceeding 50%, whereby the latter fulfilled the criterion for postoperative AKI.

The degree of the elevation differed depending on the urine creatinine concentration measured before the operation. Patients with high (≥11.3 μmol/L) urine creatinine before surgery showed a median rise in plasma creatinine of 21%, both at 6 h after surgery and on the next day, while those with low urine creatinine <11.3 μmol/L had a much smaller rise (Table 2, middle).

**Table 2:** Measurements performed on the patients during the perioperative period.

**Variable**	**U-creatinine before surgery (mmol/L)**		**Significance**
	**<11.3 (*N* = 111)**	**≥11.3 (*N* = 70)**	
**During surgery**			
Crystalloid fluid (L)	1.30 (1.00–2.00)	1.45 (1.1–2.2)	*P* = 0.56
Albumin 20% (% given)^*^	21	17	*P* = 0.47
Operating time (h)	243 (168–329)	254 (193–300)	*P* = 0.78
Blood loss (L)	0.35 (0.20–0.70)	0.40 (0.20–0.75)	*P* = 0.57
Erythrocytes received (*N*)	5	1	*P* = 0.07
**Elevation of plasma creatinine**			
To 6 h after surgery (μmol/L)	10 (−1 to 21)	21 (12–34)	*P* < 0.001
To 1 day after surgery (μmol/L)	7 (−4 to 18)	21 (6–40)	*P* < 0.001
To 2 days after surgery (μmol/L)	2 (−10 to 14)	11 (−4 to +30)	*P* < 0.02
Incidence 1 day after surgery (ratio)			
>1.25 (*N*, %)	21 (19%)	29 (41%)	*P* < 0.002
>1.50 (*N*, %)	11 (10%)	11 (16%)	*P* = 0.27
**Δ** **From preoperative to 6 h**			
Δ S-osmolality (mosmol/kg)	2 (−1 to +4)	0 (−1 to +2)	*P* < 0.002
Δ U-creatinine (mmol/L)	+3.0 (−0.1 to+7.0)	−2.5 (−6.8. to +2.5)	*P* < 0.001
Δ U-specific weight	0.009 (0.004–0.014)	0.002 (−0.002 to 0.009)	*P* < 0.001
Δ U-osmolality (mosmol/kg)	172 (18–277)	25 (−96 to +133)	*P* < 0.001

*Data are the median (25–75th percentiles) and groups compared by using the Mann-Whitney U test, except for incidences, which were compared by contingency table analysis*.

* *Median volume was 100 mL in both groups*.

Table 3 shows the preoperative urine and plasma creatinine concentrations in terms of the increasing degrees of perioperative elevations of plasma creatinine.

**Table 3:** The preoperative urine concentrations of creatinine for increasing degrees of perioperative elevations of plasma creatinine.

**Plasma creatinine Ratio 1 day after/before surgery**	**U-creatinine before surgery (mmol/L)**			
	***N***	**All patients**	***N***	**P-creatinine < 100 (μmol/L)**
<1.0	46	8.6 (6.0–11.2)	33	8.4 (5.4–10.4)
1.0–1.25	82	9.3 (6.7–13.4)	63	10.3 (7.2–14.0)
1.25–1.50	28	12.1 (9.4–15.3)	22	12.2 (11.0–15.5)
>1.50	22	10.8 (5.2–15.2)	14	11.9 (7.9–18.3)
Kruskal-Wallis test		*P* < 0.02		P < 0.0014

### Different Time Courses

Patients with low urine creatinine (<11.3 μmol/L) before surgery concentrated their urine during the surgery; all three biomarkers of urine dilution increased (creatinine, osmolality, and urine-specific weight).

By contrast, no similar concentration process occurred among the patients with a high urine creatinine prior to surgery (Table 2, bottom).

### Exploratory Analyses

The association between postoperative elevations of plasma creatinine and concentrated urine was valid only for those who had a normal plasma creatinine preoperatively (cf. Figures 1C,D).

The overall statistics did not change upon removal of the 49 patients with plasma creatinine >100 μmol/l before surgery started.

An exploratory analysis was also made after excluding the 56 patients in whom the surgery in any way involved the kidneys. The main result remained; the increase in plasma creatinine on the 1st postoperative day increased by 7 (1–15)% in those with a low urine creatinine as compared to 15 (4–24)% in those with high urine creatinine before surgery (*P* < 0.04).

## Discussion

The over-arching goal of our research is to increase knowledge about why elevation of plasma creatinine is common in the perioperative period. Approaches should be developed that can separate innocuous elevations from kidney injury of relevance to long-term prognosis. In the present study, we suggest a new mechanism that might promote elevation of plasma creatinine without necessarily involving harm to the kidney cells. The evidence can be summarized in the following four points.

### Main Findings

1. Concentrated urine before initiation of surgery was associated with a 2–3 times greater increase in plasma creatinine during the postoperative follow-up after major surgery. This is a novel finding.
2. The preoperative urine osmolality and the urine specific weight values confirmed that patients with high urine creatinine had more intense renal water conservation before surgery than those with urinary creatinine of <11.3 μmol/L, which was our criterion for regarding the urine as diluted or concentrated. Their Hb level was also 7% higher in the high urine creatinine patients, which is compatible with kidney retention of fluid due to a low daily intake of water (16).
3. The fact that plasma creatinine had increased even by 6 h after the end of surgery further suggests that the changes in urine variables were occurring during the actual operation.
4. Patients with high a urinary creatinine before surgery did not further concentrate of their urine during perioperative period. By contrast, those who had low urine creatinine showed intensified renal water concentration.

### Water Conservation During the Surgery

The urine normally undergoes a gradual concentration process during anesthesia and surgery, in particular when the urine flow rate falls below 1 mL/min (17). The reason seems to be that the low arterial pressure induced by general anesthesia inhibits the diuretic response to infused crystalloid fluid (18).

In the present study, a concentration process was evident only in patients with low urine creatinine before surgery. Those patients showed the expected pattern of changes, which consist of increases of the urine specific weight, osmolality and creatinine concentration. By contrast, very small increases in urine osmolality and urine specific weight were recorded in the patients who entered the surgery with concentrated urine, and their urine creatinine even decreased by ~20%. Thus, the kidneys concentrated the urine poorly and, in particular, could not further concentrate creatinine. This combination had a statistically significant relationship with the fact that the urine was already concentrated before the surgery started.

### Proposed Mechanism for Creatinine Elevation

In everyday life, the kidneys concentrate the urine by a factor of ~100, which is fairly close to the maximum capacity even for the normal kidney. How much further the kidneys are able to concentrate the urine is likely to depend on the individual, but the renal threshold is probably 15–25 mmol/L. The fluid retention that occurs during surgery could then be sufficient to exceed the maximum capacity to excrete creatinine, whereby creatinine accumulates in the blood. Patients with preoperatively concentrated urine may even reach the renal threshold more easily if the fluid administration program is overly restrictive; this is also a known downside of restrictive fluid therapy (3, 7).

The perioperative elevations of plasma creatinine we recorded are sufficient to make the diagnosis of AKI in 12% of the patients (1). The alternative KDIGO criterion, based on an absolute instead of a relative increase of plasma creatinine, identified virtually the same patients. Concentrated urine before surgery is a relevant factor for these elevations, although the influence seems to be moderately strong or unimportant in patients with already impaired kidney function. Inter-individual differences in the renal threshold for creatinine excretion probably confound its statistical significance. Therefore, concentrated urine and a slightly reduced renal tubular concentrating capacity before surgery may be factors that contribute to the frequent transient and harmless elevation of plasma creatinine that frequently occurs after surgery. This is a hypothesis that needs to be validated in clinical studies, as the demonstrated statistical associations we present do not infer causal relationships. However, a challenging aspect is that the hypothesis gives no reason to believe that damage to kidney cells occurs during the perioperative period in patients with transient elevation of plasma creatinine.

### Literature

A retrospective analysis of 27,860 colorectal surgery patients found an association between the preoperative blood urea nitrogen to creatinine ratio and AKI (19). Ellis et al. measured urine conductivity and found an increased risk of AKI after nephrectomy (20). Small prospective studies have suggested that concentrated urine measured as high urine osmolality, specific weight and urine creatinine may increase the risk of plasma creatinine elevation after surgery (21, 22). The largest study comprised 88 patients with abdominal cancer and reported that the 15 patients with a minor postoperative increase in plasma creatinine also had a higher body weight and more concentrated urine than the others. They had lower urine flow but a higher creatinine clearance than the other patients (mean 155 vs. 80 mL/min) during the 1st postoperative h (21). The fact that the kidneys seem to work at the peak of their ability to excrete the accumulated creatinine is compatible with the view that moderately large elevations of plasma creatinine after surgery do not necessarily imply that kidney cells have been injured.

### Limitations

There is very little mechanistic knowledge the serves to explain why plasma creatinine frequently becomes elevated in the perioperative setting. Statistical links have usually been found only to personal traits. In the present study, a high body mass index and smoking were identified as such personal risk factors.

The main part of our study focuses on short-term perioperative changes in filtering capacity of the kidneys. The focus was on *why* plasma creatinine increases rather than on the AKI diagnosis *per se*. Limitations include that health conditions that may cause long-lasting elevations of the urine creatinine concentration were not studied. Moreover, specific biomarkers of kidney injury were not measured. Several types of urologic operations were included, although our exploratory analysis suggested the contribution of urological operations did not affect the main conclusions. No long-term follow-up of the patients was made.

With regard to the preoperative situation, concentrating the urine is the body's “first line” defense to maintain the body fluid volumes when the daily intake of water is low. Volunteers with concentrated urine have stable hemodynamics when performing the “passive leg raising test” (16) and hypovolemia of clinical importance is likely to develop only very late in a chronic dehydration process.

We cannot rule out that hypovolemia during the surgery elevated plasma creatinine, but we find it unlikely due to the meticulous protocol for the clinical management of hemodynamics and fluid balance used. Invasive arterial pressure was monitored, and all patients underwent the same standard procedure with additional fluid administration and vasopressor treatment to combat hypovolemia and hypotension whenever needed. Moreover, hypovolemia and fluid deficiency would stimulate release of vasopressin that concentrates the urine, but further concentration of the urine did not occur during the perioperative period in the patients who had urinary creatinine ≥11.3 mmol/L before surgery started.

Our original study plan was to use the urine specific weight measured on the day before the surgery as the reference. However, recent research shows that a spot sample taken mid-day is an uncertain measure of the fluid balance; the optimal approach is to sample urine in the early morning or else to make a 24 h collection (6, 23). Therefore, we decided to use the urine sample collected in the early morning on the day of surgery as the reference. Urine creatinine was chosen as the key biomarker of interest because this molecule is used to diagnose AKI.

## Conclusions

A high urine concentration of creatinine (≥11.3 mmol/L) before surgery increased the incidence of a postoperative rise in plasma creatinine of 25% or more from 19% to 41%. Preoperative concentrated urine should therefore be considered a risk factor for postoperative elevation of plasma creatinine.

## Data Availability Statement

The original contributions generated for this study are included in the article/Supplementary Material, further inquiries can be directed to the corresponding author/s.

## Ethics Statement

The studies involving human participants were reviewed and approved by Ethical Committee of the Canton Bern, Switzerland (KEK Bern, Project-ID 2018-01804, Chairperson Professor C. Seiler on December 3rd, 2018). The patients/participants provided their written informed consent to participate in this study.

## Author Contributions

PW planned the study after a concept designed jointly with RH. DE, LL, and PW collected the data. RH made the calculations and wrote the manuscript, which was approved by all authors. All authors contributed to the article and approved the submitted version.

## Conflict of Interest

The authors declare that the research was conducted in the absence of any commercial or financial relationships that could be construed as a potential conflict of interest.

## Abbreviations

AKI: acute kidney injury
KDIGO: Kidney Disease Improving Global Outcome
ROC: Receiver Operating Characteristics.
